# Insights into cellular behavior and micromolecular communication in urothelial micrografts

**DOI:** 10.1038/s41598-023-40049-0

**Published:** 2023-08-21

**Authors:** Nikolai Juul, Oliver Willacy, Doste R. Mamand, Samir El Andaloussi, Jesper Eisfeldt, Clara I. Chamorro, Magdalena Fossum

**Affiliations:** 1 Laboratory of Tissue Engineering, Rigshospitalet, Faculty of Health and Medical Sciences, University of Copenhagen, Henrik Harpestrengs Vej 4C, 2100 Copenhagen, Denmark; 2 Division of Pediatric Surgery, Department of Surgery and Transplantation, Copenhagen University Hospital Rigshospitalet, Copenhagen, Denmark; 3 Department of Laboratory Medicine, Karolinska Institutet, Stockholm, Sweden; 4 Department of Clinical Genetics, Karolinska University Hospital, Stockholm, Sweden; 5 Laboratory of Tissue Engineering, Department of Women’s and Children’s Health, Karolinska Institutet, Stockholm, Sweden

**Keywords:** Urogenital models, Biomedical engineering, Paediatric urology, Translational research

## Abstract

Autologous micrografting is a technique currently applied within skin wound healing, however, the potential use for surgical correction of other organs with epithelial lining, including the urinary bladder, remains largely unexplored. Currently, little is known about the micrograft expansion potential and the micromolecular events that occur in micrografted urothelial cells. In this study, we aimed to evaluate the proliferative potential of different porcine urothelial micrograft sizes in vitro, and, furthermore, to explore how urothelial micrografts communicate and which microcellular events are triggered. We demonstrated that increased tissue fragmentation subsequently potentiated the yield of proliferative cells and the cellular expansion potential, which confirms, that the micrografting principles of skin epithelium also apply to uroepithelium. Furthermore, we targeted the expression of the extracellular signal-regulated kinase (ERK) pathway and demonstrated that ERK activation occurred predominately at the micrograft borders and that ERK inhibition led to decreased urothelial migration and proliferation. Finally, we successfully isolated extracellular vesicles from the micrograft culture medium and evaluated their contents and relevance within various enriched biological processes. Our findings substantiate the potential of applying urothelial micrografting in future tissue-engineering models for reconstructive urological surgery, and, furthermore, highlights certain mechanisms as potential targets for future wound healing treatments.

## Introduction

Congenital malformations in the urogenital organs often require reconstructive surgical interventions early in life, however, these procedures are frequently hindered by lack of healthy graft tissue required to restore a functional anatomy. Conventional surgical strategies include the use of flaps or grafts from native tissues which are harvested from other organ systems, mostly gastrointestinal, although frequently at the expense of side effects such as strictures, infections, and even malignant transformation^1–5^. Through recent decades, various tissue-engineered grafts have been introduced, seeking to optimize surgical and postoperative treatment outcomes^6,7^. One important factor, associated with successful implantation, relates to the use of cell-laden matrices, since acellular transplants have previously appeared more prone to strictures and scar tissue formation^8–11^.

**Figure 1:**
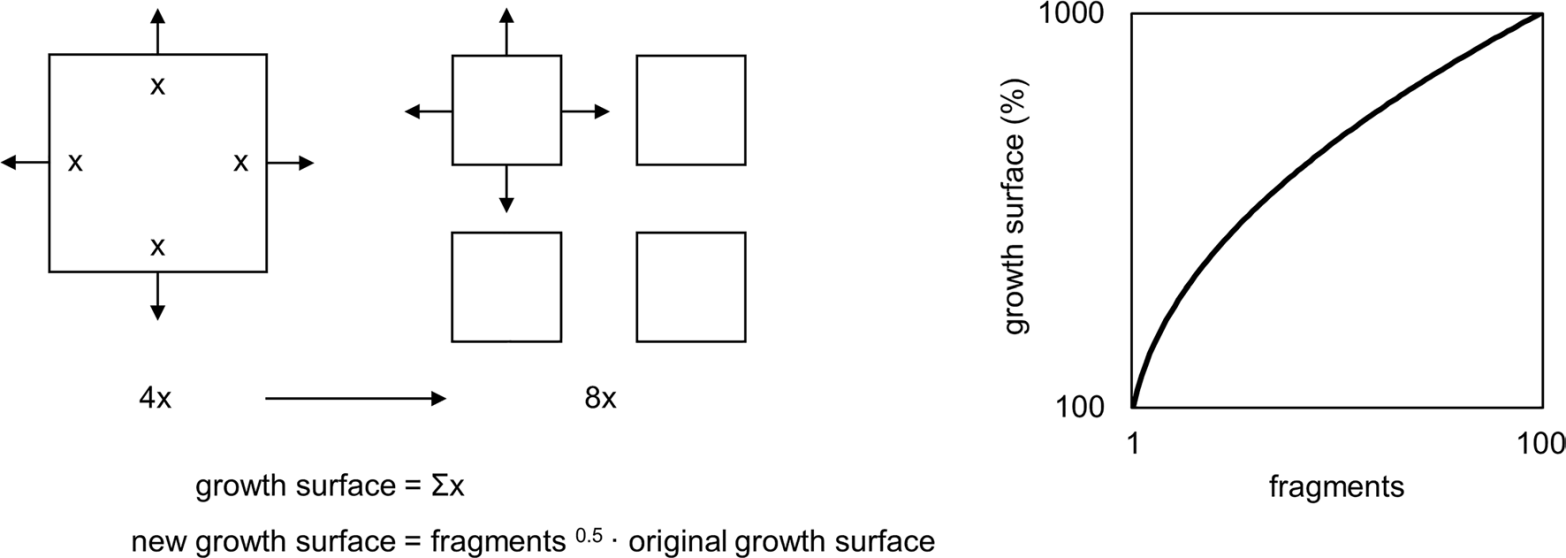
Micrograft expansion potential. Schematic illustration of the fundamental principles of micrografting according to the theory of Meek, and the numerical relationship between tissue fragmentation and expansion potential.

Autologous micrografting utilizes healthy native tissue, which is harvested and retransplanted after mechanical fragmentation. As a first proof-of-principle for micrografting, plastic surgeon Dr. C. Parker Meek reported its use for treatment of severe skin burns in a 14-year-old girl in 1958. The patient was successfully transplanted with mechanically minced native partial-thickness skin fragments (i.e., micrografts) equally distributed on her wounds. Meek hypothesized that the graft tissue expansion could be potentiated by increasing the surface area of the grafts (i.e., the micrograft wound edges), from which the keratinocytes would expand laterally^12^. Thus, the perpendicular division of a squared skin graft into four smaller fragments would potentially increase the surface area with 100%, and, accordingly, this increased growth surface area can be described with a universal formula (Fig. 1).

By these means, a small amount of graft tissue could be used to cover a larger wound area, making it a favorable option within the field of pediatric reconstructive surgery where the amount of graft tissue can be particularly scarce. In this setting, the term of *expansion ratio* refers to the proportional enlargement from the initial size of healthy graft tissue to the final grafted wound area. Meek suggested the optimal expansion ratio of micrografted skin to be 1:9 (e.g., possibly expanding the healthy graft tissue to a wound 9 times the size of the original graft), although this hypothesis has never been firmly verified^13^. In contrast, the following term of *fragmentation ratio* relates to the number of subdivided fragments of a tissue sample (e.g., a fragmentation ratio of 1:4 describes a subdivision from one piece into four smaller fragments). The micrografting technique was clinically abandoned for many years, in favor of the skin graft *meshing* technique first described by Tanner et.al. in 1964^14^. However, since the mesh grafting technique only allows for an approximate 1:5 expansion ratio, the micrografting technique has recently been broadly reappraised^15–20^. Whereas autologous micrografting has mainly been studied and applied in the treatment of skin wounds, its clinical application for other epithelium-covered organs remains largely unexplored. Furthermore, although the expansion ratio of epithelial tissue micrografts intuitively has natural upper limits, this relationship has not been fully explored and neither has it been described for uroepithelium.

The cell migration and proliferation in wounded epithelial tissue is orchestrated by complex signaling pathways, and improved knowledge on these pathways could therefore enable further development of target-specific wound healing treatments^21,22^. In previous animal models, extracellular vesicles (EVs) derived from mesenchymal stem cells have been associated with therapeutic effects regulating various phases of wound healing^23,24^. EVs are secreted by all cell types and consist of lipid bilayered structures originating from the cellular endosomal pathway. EVs carry bioactive molecules affecting paracrine intercellular communication, inducing events such as cellular migration, apoptosis, proliferation, and inflammatory responses, by releasing their contents and allowing the activation of signaling pathways in recipient cells and target organs^25^. Therefore, in the current study, we explored whether secreted EVs from the micrografts in culture specifically affected urothelial cell migration and proliferation. The extracellular signal-regulated kinase (ERK) pathway effects have mainly been linked to cell proliferation and migration in skin, however, to our knowledge, the role of the active phosphorylated form (pERK) within urothelial micrografting has previously not been evaluated^26–30^. We hypothesized, that the conditioned medium (CM) of micrografted urothelial cells contained EVs with pro-regenerative content properties. Furthermore, we hypothesized that the (ERK) pathway in urothelial cells, and the active (pERK) protein which regulates numerous intranuclear transcriptions, was affected by paracrine EV stimulation from neighboring micrografts.

**Figure 2:**
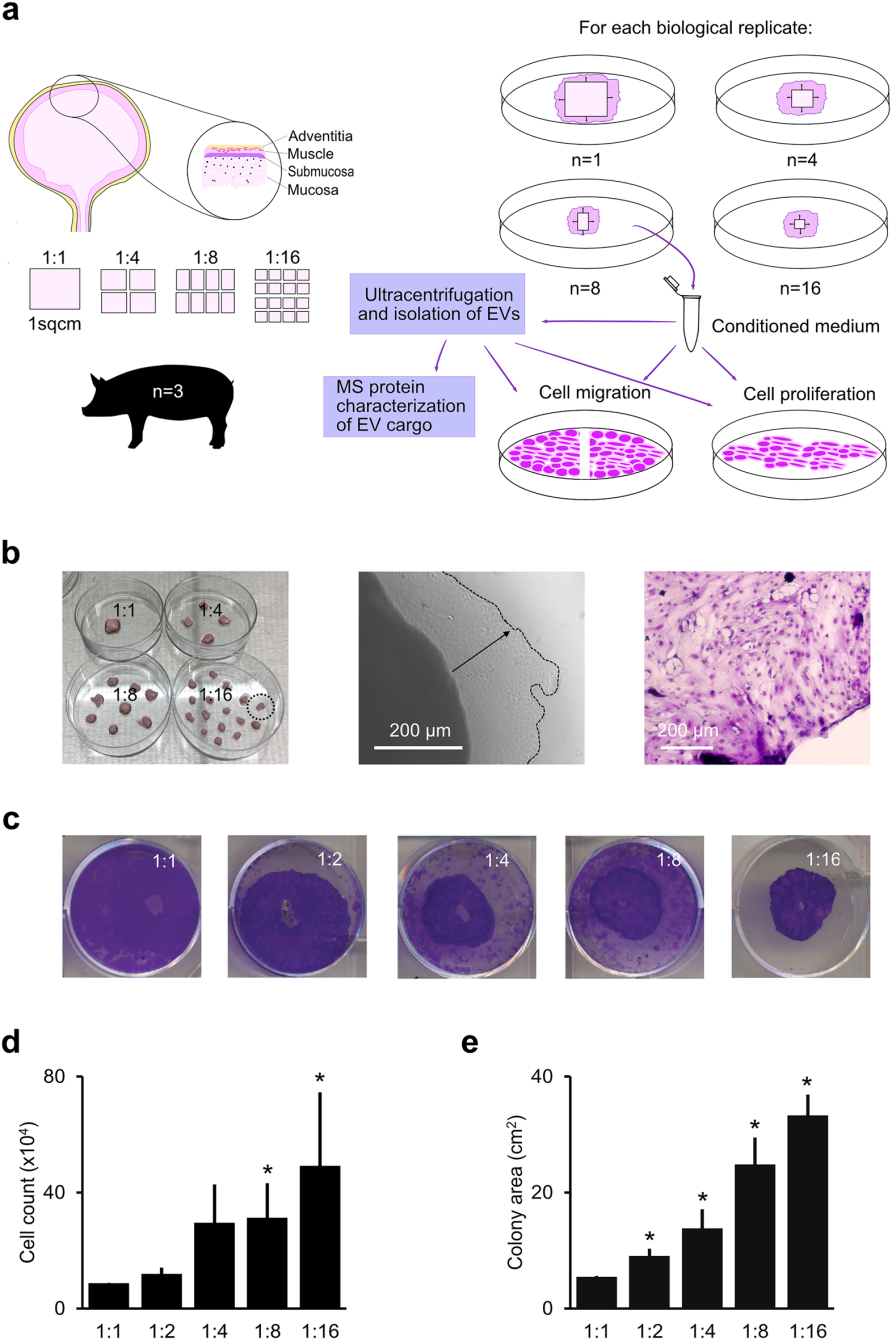
Experimental design and the relation of tissue fragmentation and cell expansion. (**a**) Schematic study representation and total cell colony quantification. Porcine bladder dissection and subdivision at different fragmentation ratios. Flowchart depicting different subsequent experiments performed from the tissue. EV: extracellular vesicle, MS: mass spectrometry. (**b**) Macroscopic image of different micrograft study conditions (left), microscopic image of urothelial cells expanding from a micrograft after 5 days (middle), and urothelial cells stained with crystal violet from a micrograft colony (right). (**c**) Examples of individual cell colony sizes from different micrograft study conditions stained with crystal violet after two weeks in culture. (**d**) Mean summative cell counts and standard deviations for each study condition after two weeks, statistically referenced to the 1:1 condition (* for p < 0.05). (**e**) Mean summative cell colony areas and standard deviations in cm^2^ for each condition after two weeks, statistically referenced to the 1:1 condition.

In this study, based on in vitro porcine urothelial micrograft cultures, our primary aim was to consolidate the theory of Meek, by establishing whether increased tissue fragmentation indeed increased the cell expansion potential also for urothelium (Fig. 2a). Furthermore, we aimed at describing the relationship between EV stimulation and ERK activation, by introducing inhibitor agents specific to the ERK pathway, in different study conditions stimulated with urothelial micrograft EVs. The biological relevance, and possible cellular signaling pathways, related to the proteins in the micrograft EVs were furthermore investigated using bioinformatic gene ontology and protein interaction analyses. We found that highly enriched ontology categories included wound healing, cellular migration, as well as ERK1/2 and Wnt signaling pathways.

## Results

### Micrograft cell colony quantification

After evaluating the propagated cell count from each micrograft study condition after seven days in culture, we found increasing numbers of proliferative cells associated with increasing tissue fragmentation ratios, (e.g., more numerous fragments from the same default tissue size yielded higher cell counts). With reference to the 1:1 study condition (one cm^2^ fragments), the halved fragments (1:2 condition) increased the cell counts with 36%, whilst the highest fragmentation ratio (1:16 condition) yielded a more than five-fold increase in cell count (563%, p < 0.05). The two study condition groups with the largest fragmentation ratios (1:8 and 1:16) yielded the highest increase in cells and were also both statistically significant (Fig. 2b,d). In line with these results, we observed significantly increased summative cell colony sizes propagated from micrografts. The relative increment in summative cell colony area increased from 66.5% (1:2 condition, p < 0.05) to 511.3% (1:16, p < 0.05) (Fig. 2c,e). In summary, we concluded that increasing fragmentation ratio consequently also increased the potential expansion ratio, indicating that the theory of Meek is indeed applicable to urothelial micrografts as well.

### Paracrine micrograft communication and ERK signaling

**Figure 3:**
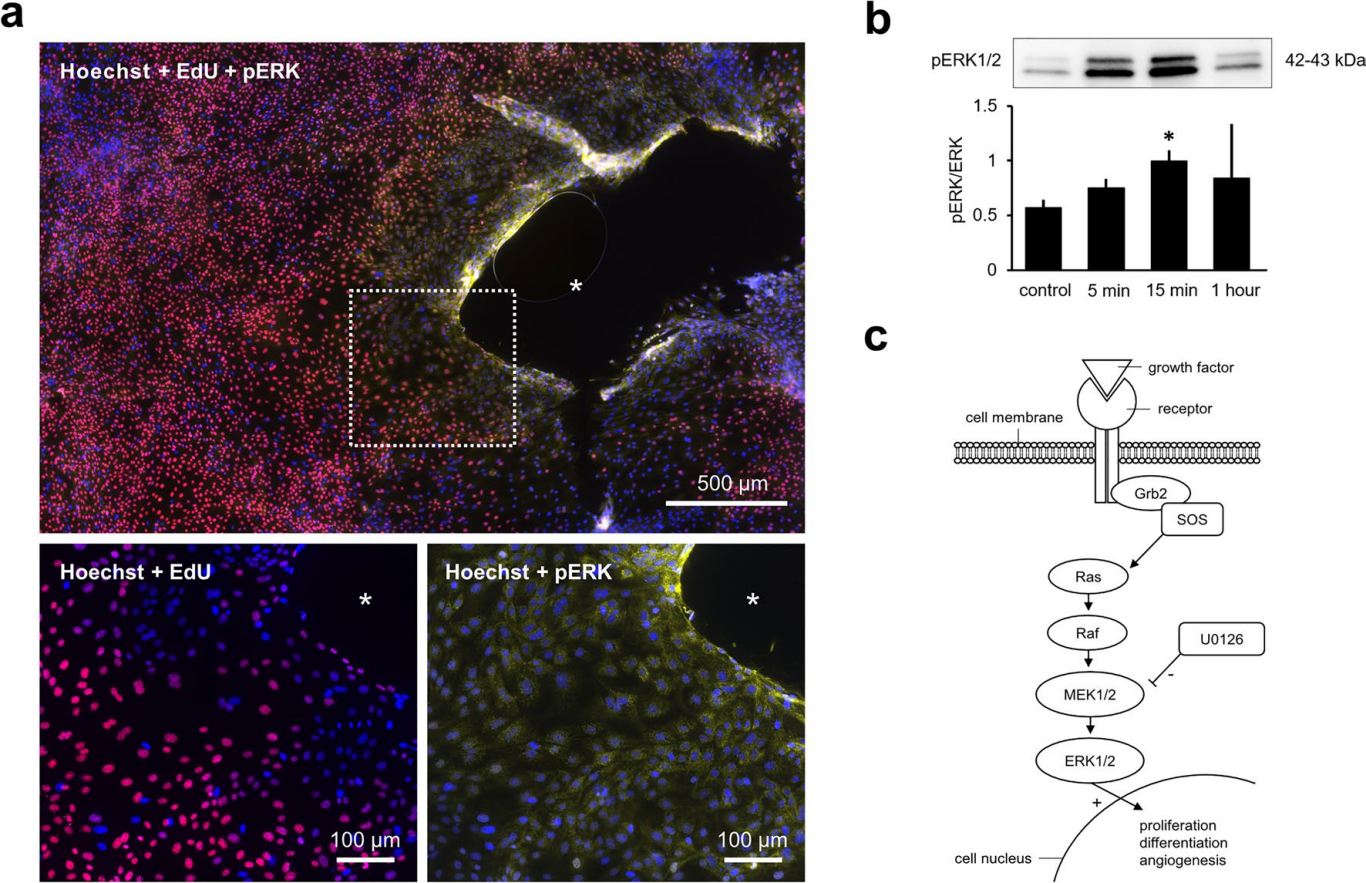
ERK expression in urothelial micrograft cultures. (**a**) Immunofluorescent stains of a urothelial micrograft colony stained with: Hoechst® 33342 cell nucleic marker (blue), EdU (5-ethynyl-2’-deoyuridine) coupled with Alexa Flour® (picolyl azide) as a proliferative marker (red), and pERK (phosphorylated extracellular signal-regulated kinase) antibody (yellow). (**b**) Normalized expression of pERK (phosphorylated extracellular signal-regulated kinase) proportional to the expression of total ERK at three timepoints after stimulation with conditioned culture medium, statistically referenced to the stimulation with fresh keratinocyte culture medium used as control. Examples of cropped bands from the Western blots analyzed at different timepoints after stimulation with conditioned medium and with fresh medium as the control represented above (original blots are presented in Supplementary Fig. S1). (**c**) Schematic representation of the ERK signaling pathway; *SOS* son of sevenless guanine nucleotide exchange factor, *Grb2* growth factor receptor-bound protein 2, *MEK1/2* mitogen-activated protein kinase kinase 1 and 2, *ERK1/2* extracellular signal-regulated kinase 1 and 2, *Ras* Ras GTPase, *Raf* Raf kinase kinase, *U0126* 1,4-diamino-2,3-dicyano-1.4-bis[2-aminophenylthio]butadiene.

After evaluating immunofluorescent stains of urothelial micrografts, we observed that the expression of pERK was primarily located at the borders of the micrografts, whereas the EdU-positive (5-ethynyl-2ʹ-deoyuridine) proliferative cells were generally located in less crowded areas more distal from the micrografts (Fig. 3a). By Western blot, the levels of pERK expression in cell cultures stimulated with micrograft conditioned medium (CM) (for either 5 min, 15 min, or 1 h) were all elevated compared to controls (stimulation with fresh growth medium). The levels of pERK (normalized to total ERK levels) reached a maximum after approximately 15 min of stimulation, at which time the pERK levels were also significantly increased compared to the control (pERK/tERK ratio of 0.93 vs. 0.52, respectively, p < 0.05) (Fig. 3b,c).

### EV isolation and quantification

**Figure 4:**
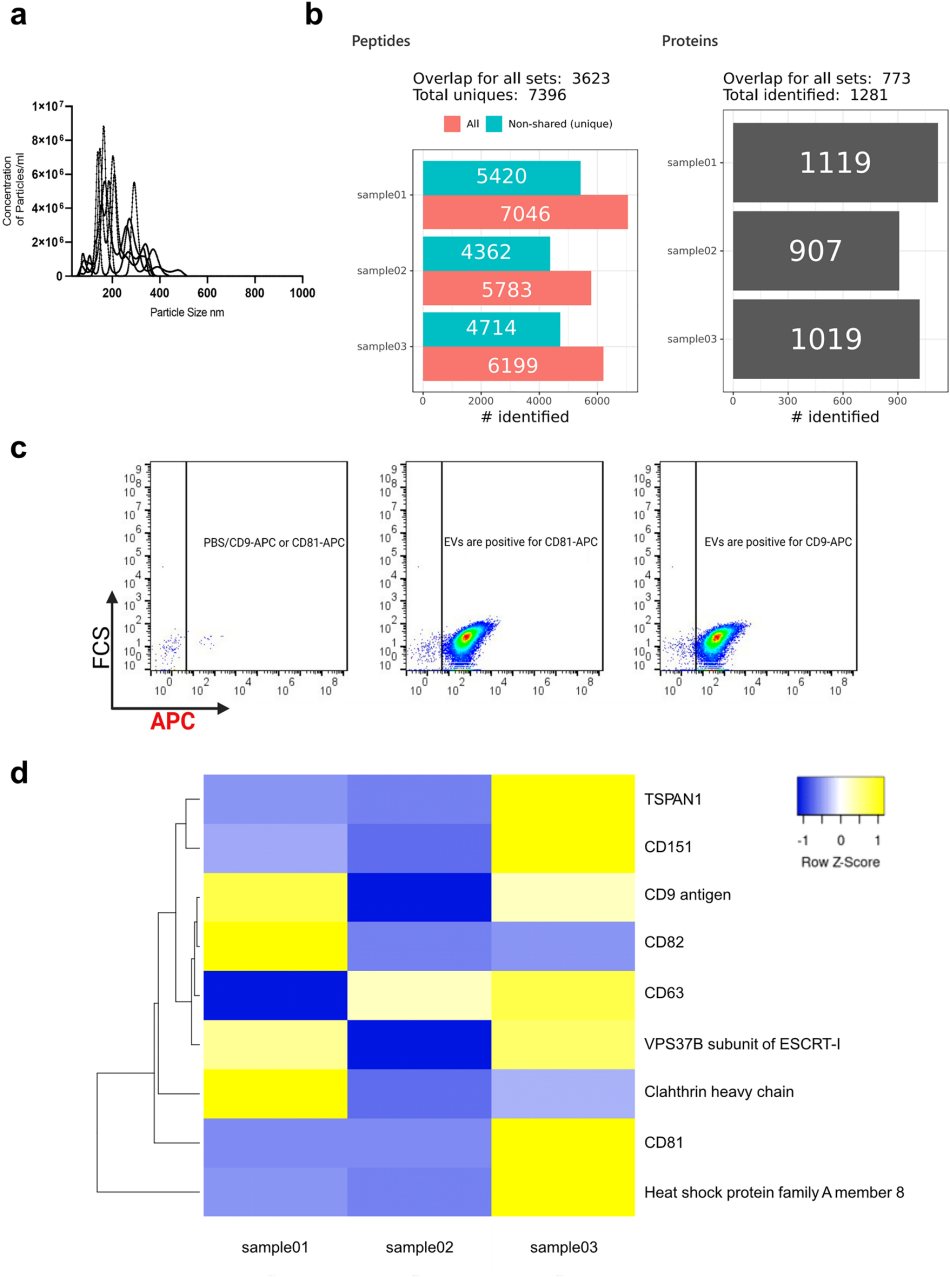
Isolation and characterization of micrograft extracellular vesicles. (**a**) Nanoparticle tracking analysis was used to measure the size and concentration of the particles. (**b**) Mass spectrometry summary of the EV cargo and summary of total number of identified peptides and proteins. (**c**) EVs markers were detected according to forward scatter signal (FCS) using high-resolution imaging flow cytometry (Amnis CellStream) and tetraspanin-labeled-allophycocyanine (APC) anti-Big. (**d**) Heat map representation of EVS markers identified by MS; the intensity represents Signal intensity of the precursor peptides identified. Clustering based on average linkage and Euclidean distance measurement.

Using tangential flow filtration (TFF) following size exclusion chromatography (SEC), we isolated EVs from micrograft cultures. We then proceeded to characterize the size profiles of the total amount of isolated particles using nanoparticle tracking analysis (NTA). The findings indicated that the particle sizes were highly heterogeneous, displaying small and large particles between 50 and 350 nm. This size range is characteristic of EVs, such as exosomes and microvesicles, respectively (Fig. 4a). Proteomic analysis results identified approximately 1280 proteins from isolated EVs (Fig. 4b). To further characterize the particles, we used highly sensitive flow cytometry to detect EV markers, including tetraspanins (CD9, CD63, CD81, and CD82). The data indicated that the EVs derived from micrograft cultures were positive for both of the common EV surface markers CD9 and CD81 (Fig. 4c).

Finally, EV-specific extracellular markers such as tetraspanins, Clathrin and VPS37b, and Heat-shock proteins, all of them multivesicular body associated proteins, were clustered according to their prevalence across the experimental replicates (Fig. 4d).

### Functional evaluations of CM and EV stimulation

**Figure 5:**
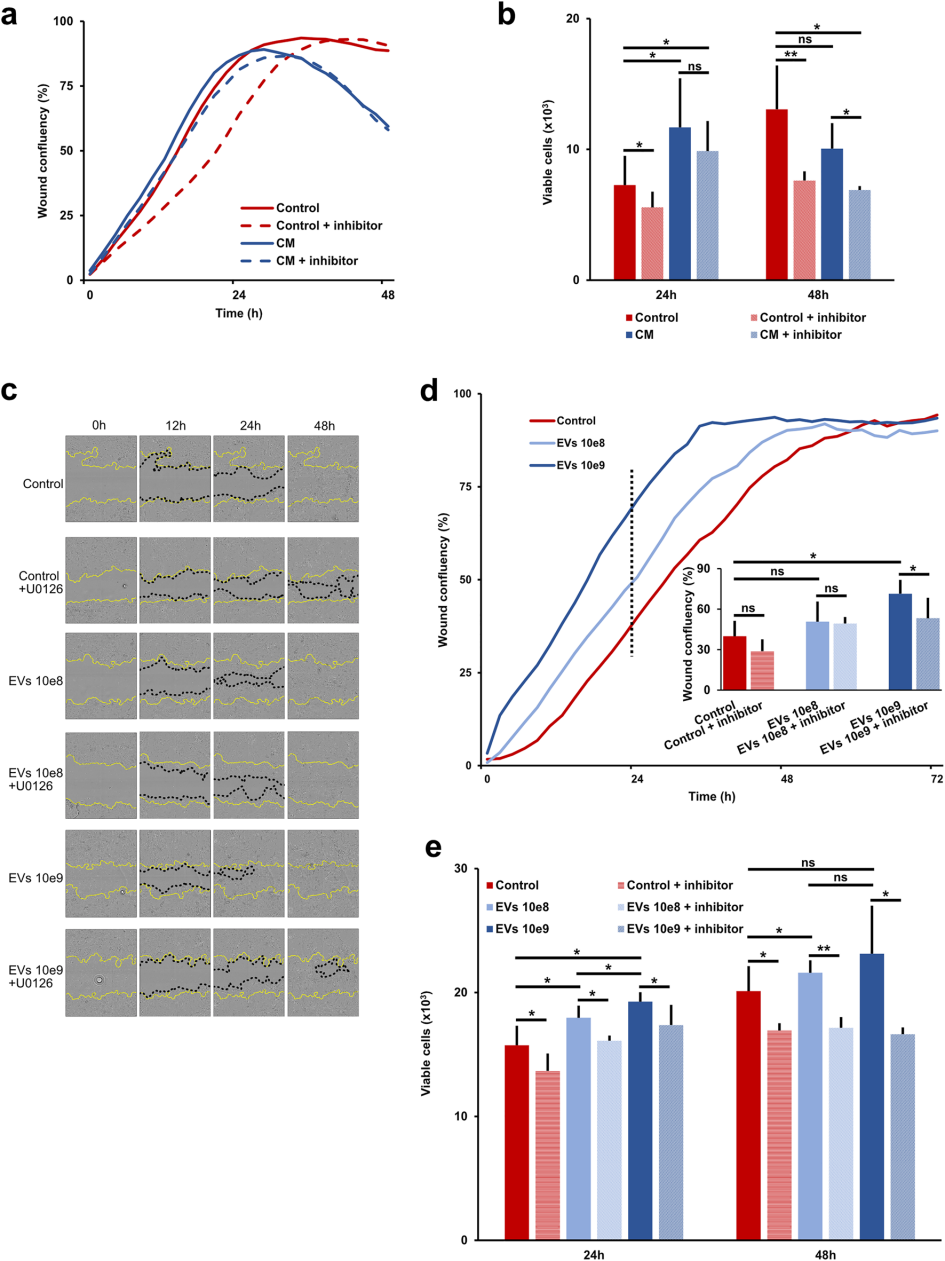
Stimulatory effects of micrograft extracellular vesicle contents. (**a**) Wound confluency rates of monolayered urothelial cell cultures after standardized scratching and stimulation with either fresh culture medium (control) with or without ERK inhibitor (+ inhibitor) or conditioned culture medium (CM) with or without inhibitor. (**b**) Mean number and standard deviation of monolayered urothelial cells per 20 mm^2^ after 24 and 48 h of stimulation with either CM or control medium, with or without additional inhibitor (* for p < 0.05, ** for p < 0.0001 and ns: not significant). (**c**) Examples of wound confluency at different timepoints after wounding and stimulation with control medium with either additional EVs at 10e8 or 10e9 µM concentration and with or without additional ERK inhibitor, respectively. (**d**) Wound confluency rates of monolayered urothelial cell cultures after standardized scratching and stimulation with either control or different concentrations of EVs and with or without additional inhibitor. Compared mean wound confluency (%) with standard deviations 24 h after wounding (lower right). (**e**) Mean number and standard deviation of monolayered urothelial cells per 20 mm^2^ after 24 and 48 h of stimulation with either control or different concentrations of EVs and with or without additional inhibitor (* for p < 0.05, ** for p < 0.0001, and ns: not significant).

We further evaluated the functional effects of cell culture stimulation with control vs. CM, and, furthermore, the additional effect of a specific upstream inhibitor (U0126 acting on MEK1/2) for the ERK signaling pathway (Fig. 3c). From scratch assays, we found tendencies of increased migration in cells stimulated with CM compared to control, and an inhibiting effect if adding the ERK inhibitor (Fig. 5a). When evaluating the number of proliferative cells in each condition, after 24 h of stimulation, cell cultures stimulated with CM contained significantly more cells compared to the control group (11.68 × 10^3^ vs. 7.26 × 10^3^ cells/well, p < 0.05). While the addition of ERK inhibitor to the CM group did not significantly decrease the number of proliferative cells, a significant decrease was, however, encountered after inhibitor addition to the control group (5.56 × 10^3^ cells/well with inhibitor vs. 7.26 × 10^3^ without inhibitor, p < 0.05). After 48 h of stimulation, the stimulatory effect of the CM group seemed to wear off and became significantly decreased compared to the control group (10.05 × 10^3^ vs. 13.06 × 10^3^ cells/well, respectively, p < 0.05). In both groups, inhibitor addition significantly decreased the number of proliferative cells after 48 h of stimulation (Fig. 5b).

In agreement with our initial findings, fresh culture medium with additional EVs induced a significant increase in cell migration and proliferation compared to fresh culture medium without EVs (control), and, again, this effect was partly abolished 48 h after addition of the ERK inhibitor (Fig. 5c–e).

Altogether, we found that in vitro proliferation and migration of micrografted urothelial cells was promoted through paracrine communication (as seen from the stimulatory effect of CM) and that the stimulatory effects indicatively involved the ERK pathway.

### EV proteome analysis

**Figure 6:**
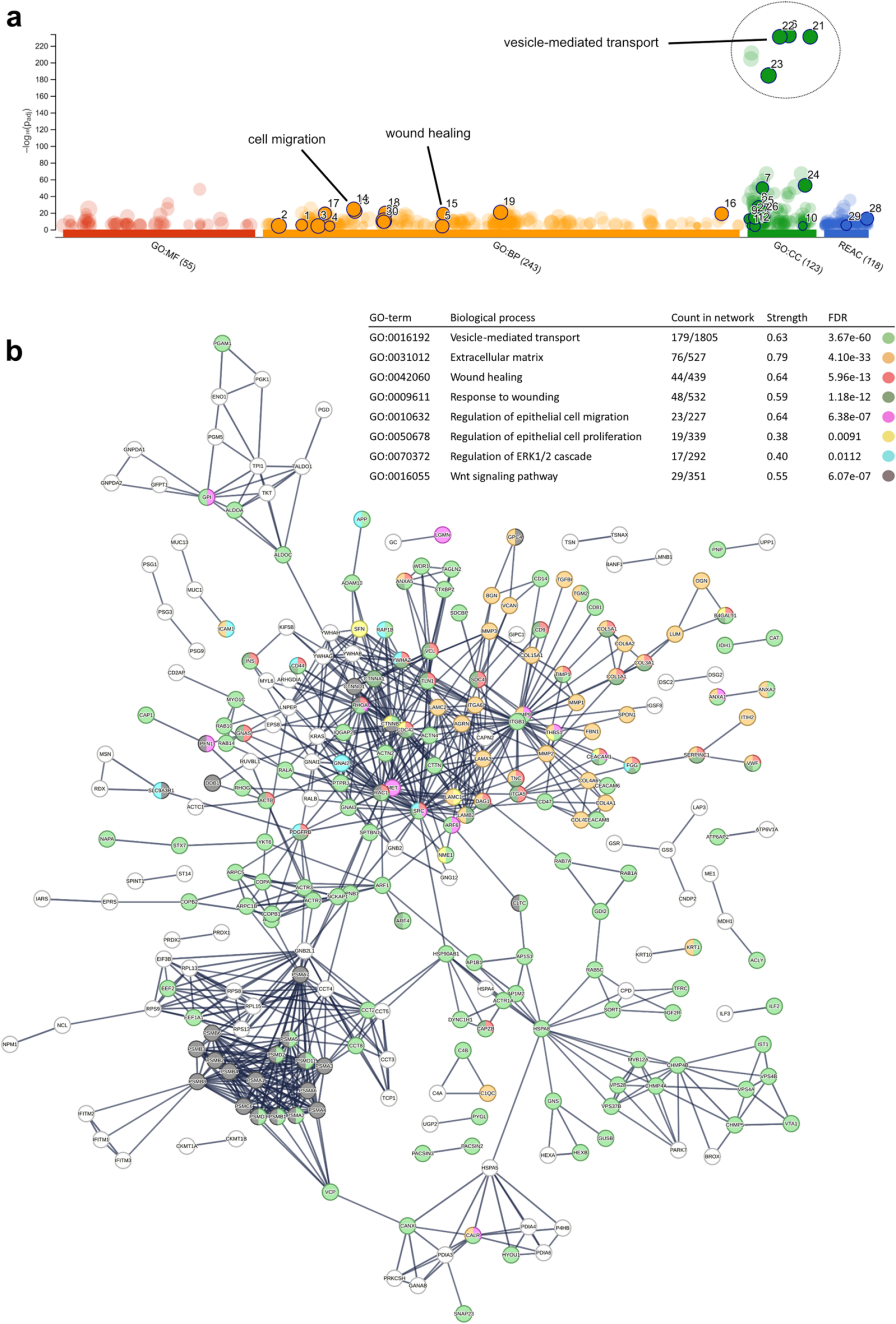
Biological characterization of the extracellular vesicle proteome. (**a**) Manhattan plot showing results from functional profiling in g:Profiler. Gene Ontology (GO) terms under the categories molecular function (MF), biological process (BP), cellular component (CC), and Reactome pathways (REAC), displayed in color coding. (**b**) A detailed result view on enriched GO terms from protein–protein interaction network generated using the STRING resource including proteins that fall into several of the GO enriched terms, with respective false detection rates (FDR).

After normalizing of the mass-spectrometry data and linkage to orthologous human genes, a gene ontology (GO) analysis was performed using the web interfaces g:Profiler and the Global Core Biodata Resource Panther. The list of EV isolated proteins resulted in significant enriched Gene ontologies (FDR and P-value both < 0.05). As expected, we found several highly enriched pathways related to vesicle-mediated transport, but also related to cell migration and wound healing (Fig. 6a)^31,32^. Enriched GO terms in the categories of cellular components, biological processes, and molecular functions included terms such as wound healing, cell differentiation, cell migration, vesicle-mediated transport, and extracellular matrix. Pathway enrichment analysis, furthermore, identified several overrepresented signaling pathways including ERK1/2 regulation, and, interestingly, enriched clusters related to the Wnt pathway (cut-off criterion of significant enriched REACT pathways was P < 0.0001) (Fig. 6b). All identified EV proteins used in the pathway enrichment analyses can be found in the Supplementary Table S1.

## Discussion

In this study, we investigated how micrograft fragmentation potentiated the cell migration and proliferation of porcine urothelial tissue in vitro. With reference to a one cm^2^ mucosal tissue micrograft, we demonstrated that further tissue fragmentation yielded a significant increment in both cell proliferation and cell colony expansion area. Furthermore, we explored whether the ERK pathway was specifically involved in these processes and demonstrated that specific inhibition of the pathway significantly reduced both cell migration and proliferation in vitro. We extracted conditioned culture medium from micrograft cell cultures and, subsequently, we used the extracted medium to stimulate monolayered urothelial cell cultures, which had been further propagated from the same primary cell line. In these experiments, we observed that the conditioned culture medium contained extracellular contents with stimulatory effects on both urothelial cell migration and proliferation, and that these effects could be partly reversed by specifically inhibiting the ERK pathway. The activation of the ERK pathway was furthermore confirmed by localization experiments on bladder mucosal micrografts, indicating local flux of molecules within this signaling pathway at the micrograft borders. From Western blot, we observed that the phosphorylation of ERK (i.e., the active form) reached a maximum after 15 min of stimulation with conditioned medium (and was since normalized after one hour). Since our results from our initial experiments indicated that the CM was depleted of nutrients after the first 24 h (e.g., as seen in the deteriorating cell confluence 48 h after scratch wounding), we decided to isolate EVs from the CM and add to fresh culture medium in repeated experiments. From repeated functional in vitro experiments, we concluded that the isolated vesicular contents reproduced the stimulation of both migration and proliferation, as seen in the previous experiments, and that, again, the effects could be regulated by inhibiting the ERK pathway activation. Finally, we characterized the EV protein contents by mass spectrometry and applied bioinformatic analysis to identify associated enriched biological pathways of particular relevance to the study settings.

Although our findings indicate an intricate relation between the stimulatory effects of secreted extracellular vesicles and the activation of the ERK pathway, larger and more advanced experimental models would be required to finally confirm this hypothesis. Our findings do, however, correspond well with those recently reported by Balli et al., when applying a similar wound healing skin model in rodents to study micrografted keratinocytes^33^. In their study, the important role of the ERK pathway on cell migration was confirmed both in vitro and in vivo. Interestingly, however, the authors did not observe significant ERK-related effects on cell proliferation, whereas we observed a significant negative impact from upstream ERK-inhibition on cell proliferation. Therefore, it could be speculated whether elemental differences between the intracellular regulation of keratinocytes and urothelial cells exist, albeit this inconsistency could as well rely on unidentified experimental differences. The general ERK activation in micrografted epithelial tissues is of considerable interest on its own, however, further differentiated knowledge on the ERK-specific roles in cell regeneration is of substantial importance, since it would potentially further enable researchers to design target-specific treatments, directly applicable in future wound healing therapies. Whereas ERK activation has previously been linked to an increased migratory rate in urothelial cells, the specific cellular migratory patterns from urothelial micrografts have not previously been studied^34,35^. Even though our study highlighted specific secreted proteins and activated biological pathways relevant for micrografts and wound healing in general, this part of our study was performed exploratively on a small-scale basis, and, consequently, we are currently only scratching the surface of the micromolecular events governing micrograft regeneration. Nevertheless, our findings have led us to pursue further experiments, in larger settings and with additional control groups, to further unravel these mechanisms and more extensively quantify specific EV contents which are unique to the micrografted cells.

This study is, to our knowledge, the first study to characterize the micromolecular effects of micrografted urothelial tissue, although under highly controlled laboratory conditions. In this perspective, Strehl et al. previously described how conventional in vitro cell culture environments, similar to the current study, can potentiate the mitotic cell phases and hereby possibly skew cell differentiation compared with an in vivo setting^36^. Micrografts have previously been studied mainly in skin tissue, however, the introduction of urothelial cells to the concept poses another possible challenge: whereas skin epithelium is exposed to atmospheric air and ex vivo mechanical manipulation, the native surface environment of epithelium in hollow organs differs substantially. Therefore, we do not know whether for example in vitro culturing in a urine-liquid interface would possibly alter our results. Finally, despite that both biological and technical sample triplicates were ensured for each study condition, the unprecedented nature of this study did not enable us to perform complete prespecified sample size calculations, and, therefore, our results may have been influenced by underpowered data.

The concept of micrografting is of particular importance within tissue engineering, as an accessible and simple method for cellularizing scaffolds before or during surgical grafting. Although tissue engineering studies have introduced several promising reconstructive surgical treatment options, relevant in congenital malformation surgery among others, numerous biocompatible scaffolds have previously failed in clinical or preclinical trials, most often due to either shrinkage or stricture formation^37,38^. It is widely agreed, that one reason for failure relates to the absence of scaffold-seeded cells, since the repopulating of tissue-engineered matrices is believed to counteract the formation of scar tissue^8^. Furthermore, in a future clinical setting, it will be imperative to ensure a safe standard of healthy harvesting autologous tissue before surgical reimplantation, and this aspect would indeed represent a challenge for the current applicability of our construct in oncological cases. For these cases, other tissue sources would be needed^39^.

In our own previous studies, we have demonstrated that urothelial micrografts, from both pigs and humans, can be successfully expanded on an easily applicable polymer-enforced collagen-based scaffold, both in vitro and more recently in vivo^40–44^*.* Since the construction of cell-seeded tissue engineered scaffolds is often challenged by the need for pre-implantation ex vivo cell propagation, requiring resource-intensive facilities, our goal was to develop an easily available and single-staged procedure for the fabrication and implantation of a transplant. In this context, autologous micrografts, harvested perioperatively, represents an intelligible solution for loading the scaffold for in vivo cellularization. Therefore, in continuation of our studies, it was important to understand the basic behavior and function of the micrograft components of our scaffold before embarking further in vivo experiments. A main finding in this study, is the induced increment in cell expansion potential demonstrated by increased tissue fragmentation. This finding has encouraged us to continue applying the micrografting technique as a component in ongoing tissue-engineered models for urogenital reconstruction. Nevertheless, we believe that the hypothesis of favorable in vivo outcomes from cellularized scaffolds continuously demands further challenging in larger preclinical in vivo studies. Furthermore, the application of EVs in tissue-engineered scaffolds is a field of future potential. As an example, Xu et. al. recently demonstrated how EVs, derived from human urine-derived stem cells and infused in hydrogel-based implants for in vivo rabbit vaginoplasty, induced significantly increased epithelialization and angiogenesis, and, furthermore, upregulated the intracellular ERK1/2 phosphorylation^24^.

In conclusion, we confirmed that increased tissue fragmentation does potentiate the regenerative properties and the expansion potential of urothelial micrografts. Furthermore, we observed positive indications that micrografted urothelial cells are capable of excreting pro-generative substances, which merit further studies on specific target identification for new wound healing therapies. Our findings have encouraged us to continue applying micrografts as a component in the scaffold-development for reconstructive implantation in in vivo studies.

## Methods

### Micrograft cell colony quantification

Urinary bladders from three full-grown female Landrace pigs were excised in toto, immediately after animal euthanasia by lethal intravenous pentobarbital injection. After harvesting the bladders under sterile conditions, the organs were transferred in sterile phosphate buffered saline (PBS, Gibco, Thermo Fisher Scientific) directly to the cell culture laboratory and, furthermore, washed three times in PBS containing penicillin and streptomycin (50 IU/ml and 100 µg/ml, Gibco). Subsequently, the urothelial layer from each bladder was dissected under sterile conditions. The urothelium was cut into 1 cm^2^ squares with a scalpel and assigned for one of the following study conditions (i.e., fragmentation ratios): 1:1 (no further fragmentation), 1:2 (halved), 1:4 (quartered), 1:8 (each quarter was halved), and 1:16 (each quarter was quartered) (Fig. 2a). Each of the epithelial fragments were then placed separately in 9.6 cm^2^ culture wells (6-well plates) with the luminal tissue side facing downwards. Consequently, each of the study conditions were replicated in biological triplicates separately for each of the following experiments described below. The culture wells were supplemented with 2 ml of customized culture medium, according to previous description^40^: Dulbecco's modified Eagle's medium + Ham’s F12, mixed 4:1, (Gibco, Thermo Fisher Scientific) supplemented with fetal bovine serum (10%, Gibco), antibiotics (penicillin 50 IU/ml and streptomycin 100 µg/ml, Thermo Fisher Scientific), hydrocortisone (0.4 µg/ml, Calbiochem, Sigma Aldrich, 386,698), insulin (5 µg/ml), triiodothyronine (1.35 ng/ml), transferrin (5 µg/ml), cholera toxin (8.4 ng/ml, Sigma Aldrich C3012), adenine (21 µg/ml, Sigma Aldrich) with addition of epidermal growth factor (10 ng/ml) after the first 24 h. The tissue fragments were cultured at 37°C and 5% CO_2_ levels, with medium change every second day.

#### Quantifying the total number of viable cells from each micrograft condition

After 14 days, the propagated cells from each micrograft study condition were trypsinated (0.5%, Sigma-Aldrich, US) and centrifugated for 5 min at 1000 rpm. The pellets were resuspended with 1 mL PBS and stained with Trypan Blue (4%, Invitrogen, Thermo Fisher, US). Next, the cells were quantified using the Countess Cell Counter™ following the manufacturer’s instructions (Invitrogen). The sum of propagated cells from each study condition in each biological replicate was obtained and compared to the respective 1:1 condition (e.g., the sum of propagated cells from all four cultured fragments from the same 1:4 study condition replicate was calculated).

#### Estimation of the total expansion area from each micrograft condition

After 14 days, the propagated cell colonies on the bottom of each culture well were fixated with 4% formalin and stained with a crystal violet solution (0.5%, Sigma-Aldrich) (Fig. 2b). Digital image scans of each colony were performed, and the total colony area was quantified using the ImageJ software (version 1.8.0_172, Rasband, W.S., U. S. National Institutes of Health, US). The summarized colony areas for each study condition in each biological replicate were evaluated and compared to the respective 1:1 study condition (e.g., the area sum of all four colonies from the same 1:4 condition replicate was calculated).

### Paracrine micrograft functions and ERK signaling

Primary pig urothelial cells were isolated from pig urothelial tissue explants (1:16 micrografts from each biological replicate) and cultured using customized culture medium (as described above in Sect. 1). When the propagated colonies reached a size of approximately half a centimeter (approximately after 1 week in culture), the conditioned culture medium (24 h old) was saved from each biological replicate, centrifugated for 10 min at 1000 rpm, filtered using a 20 µm filter (Millipore, Merck, US) and stored at – 20 °C until its use in the following proliferation and migration experiments. By these means, conditioned culture medium could be used to stimulate the same primary cell culture from which it was originally obtained, later during the experiment. The cells were cultured up to passage two before being stimulated with the thawed conditioned medium initially collected from the explants.

#### Immunofluorescent staining

One week old colonies from urothelial tissue micrografts were incubated with the cell proliferation Click-iT® Plus reaction kit as per manufacturer’s instructions (Life Technologies, Thermo Fisher, US) with EdU (5-ethynyl-2ʹ-deoxyuridine) coupled to Alexa Flour® (picolyl azide) acting as a proliferative marker. The colonies were then fixated (4% buffered formalin, VWR Chemicals) and permeabilized (0.5% Triton® 100× in PBS). Thereafter, the cell colonies were stained with Hoechst® 33342 (5 µg/ml bisbenzimide, Thermo Fisher) cell nucleic marker and pERK1/2 antibody (cat#44-680G, Invitrogen). Immunofluorescent microscopic imaging was finally obtained using the Olympus VS200 scanner slide and OlyVia™ software for further qualitative evaluation (Olympus VS200, Life science Solutions, LRI Instruments AB, SE) at the Core facility HistoCore, Karolinska Institutet.

#### Protein extraction and Western blot

Primary monolayered pig urothelial cells were cultured in fresh culture medium (control) or stimulated with thawed conditioned medium for either 5 min, 15 min, or 1 h. After stimulation, each micrograft culture well was washed with PBS and the cells were lysed using radioimmunoprecipitation assay (RIPA) buffer (150 mM, NaCl, 1.0% IGEPAL® CA-630, 0.5% sodium deoxycholate, 0.1% SDS, 50 mM Tris, pH 8, Sigma-Aldrich) supplemented with a mixture of proteinase and phosphatase inhibitors (cOmplete™ ULTRA Tablets, Mini, EASYpack Protease Inhibitor Cocktail, Roche, CH). Total protein concentration was evaluated using the bicinchoninic acid (BCA) assay as per manufacturer’s instructions (Thermo Fisher Scientific, US). Fifty micrograms of total protein was heat-denatured in sample-loading buffer (4 × Laemmli sample buffer, Bio-Rad Laboratories, US) and loaded into 7.5% SDS–polyacrylamide (SDS-PAGE) electrophoresis gel (Mini-PROTEAN® TGX™ Precast Gels, Bio-Rad) and then transferred onto polyvinylidene fluoride (PVDF) membranes (Trans-Blot Turbo Transfer System, Bio-Rad). The membranes were thereafter blocked with Tris-buffered saline (TBS) containing 5% bovine serum albumin (BSA) and incubated over night with a 1:1000 dilution of primary antibodies specific for either ERK1/2 (rabbit ERK1/ERK2 monoclonal antibody, cat#MA5-15134, Invitrogen) or pERK1/2 (rabbit phospho-ERK1/ERK2(Thr185, Tyr187) polyclonal antibody, cat#44-680G, Invitrogen). Anti-mouse secondary horseradish peroxides (HRP) conjugated antibody was thereafter used (Anti-mouse IgG HRP-linked antibody, cat#7076p2, Cell Signaling Technology, US) at a 1:4000 dilution and incubated for two hours. The chemiluminescent Clarity Max Western ECL substrate (Bio-Rad) was used for signal detection. Signal analysis was performed with the Gel Doc™ chemiluminescence detection system (Bio-Rad). Relative densitometric analysis was performed with the Image Lab software™ (version 5.2, Bio-Rad), and proportional changes in pERK/ERK and relative volumetric changes were calculated with reference to the control condition (no stimulation). Total gels used for analysis in Fig. 3 are presented in Supplementary Fig. S1.

#### Migration assay

For each biological replicate, 20 experimental replicates of primary cell cultures were plated in a 96-well culture plate with approximately 40,000 seeded cells per well. After 24 h, the cells had reached a confluent monolayer and a standardized 1 mm scratch wound was then applied in each well using a WoundMaker™ (Essen Bioscience Ltd., US). The experimental replicates were then equally assigned to one of the following treatments: stimulation with fresh culture medium with or without an additional specific ERK inhibitor (U0126 100 mM, Sigma Aldrich, US), stimulation with conditioned medium with or without inhibitor and stimulation with isolated extracellular vesicles at the indicated concentration with or without ERK inhibitor. Live-cell imaging using the IncuCyte™ system (Essen Bioscience) was then obtained over the coursing three days, and the IncuCyte Zoom software (version S3, Essen Bioscience) was used to evaluate the migratory rates and consequent wound confluency of each treatment regimen.

#### Proliferation assay

In parallel with the setup described in the previous section, 20 experimental replicates of primary pig urothelial cell cultures from each biological replicate were seeded in a 96-well culture plate, with 20,000 cells per well. Again, the experimental replicates were assigned to one of the four regimens (CM, fresh medium or isolated extracellular vesicles with or without inhibitor). After 24 h of stimulation, the number of viable cells were evaluated using the Cell Counting Kit 8™ as per manufacturer’s instructions (Sigma Aldrich) and subsequent spectrophotometric analysis using a microplate reader (Multiscan™ Sky photometer, Thermo Fisher Scientific). A standard curve was created from known concentrations of non-stimulated urothelial cells from the same primary culture, and finally used to quantify the viable cells in each of the four regimens.

### EV isolation, quantification, and proteome analysis

In these experiments, the extracellular contents of conditioned serum-free culture medium from micrografts cell cultures were extracted for proteomic analysis through ultracentrifugation, and the contents were analyzed as described in the following sections.

#### Isolation and purification of EVs

Porcine urothelial micrografts (about 2 × 2 mm^2^ in size) were planted in 15 cm Petri-dishes (Sarstedt, DE) and cultured in cell culture medium at 5% CO_2_ and 37 °C humidified incubator as indicated above. After one week in culture the urothelial cell culture was replaced serum free media Opti-MEM (Gibco) supplemented with antibiotics (penicillin 50 IU/ml and streptomycin 100 µg/ml, Thermo Fisher). After 48 h, the conditioned medium (CM) was harvested pre-cleared by centrifugation for 5 min at 700×*g* to remove cells, followed by 10 min using high speed at 2000×*g* to eliminate debris and larger particles. Prior to employing tangential flow filtration (TFF) (KrosFlo® KR2i, Repligen, US) the supernatant was further purified using 0.22 µm vacuum filtration unit (Sarstedt) to remove remaining undesired larger particles. Then TFF was employed for separation, purification, and concentration of EVs via 300 kDa cut-off hollow fibre filters at 3.0 psi transmembrane pressure. Final volume of 35 ml supernatant was collected and purified by 0.22 µm vacuum filtration. The supernatant was further concentrated, using 10 kD Amicon® ultra-15 centrifugal filter unit (Merk, Germany). The EVs were purified more by size exclusion chromatography (SEC), using 70 nm 500 µL qEV columns (qEVoriginal; IZON Science LTD, NZ), and collecting the 4th and 5th ml of elute. The elute was then concentrated to a final volume of 200–300 µL, using 10 kDa spin filters (Amicon Ultra; Millipore) spun at 4000 RCF^45^. Finally, the EVs were collected, characterized, and stored at – 80 °C in a storage buffer for later usage^46^.

#### *Nanoparticle tracking analysis (NTA*)

NTA was used to measure particle size and concentration in the samples using a NanoSight NS500 equipped with NTA 2.3 analytical software and a 488 nm laser (Malvern Panalytical, UK). 0.22 µm filtered PBS was used to dilute the samples before they were analyzed. Per sample, five 30-s videos were captured at a camera level of 14–15. For each measurement, the analytic software parameters maintained the same (screen gain 2, detection threshold 6).

#### Imaging flow cytometry analysis (IFCM)

High resolution IFCM was employed to analyze single EVs using an Amnis CellStream instrument (Luminex, DK) equipped with 405, 488, 561, and 642 nm lasers. According to the previously optimized settings and methods^47^. Before analysis, a volume of 25 µl from each sample was diluted 500 times in 0.22 µm filtered PBS buffer. As previously described^47^; Briefly, all aliquots of EVs were incubated with APC-labelled anti-CD81 (ThermoFisher, cat# 17-0819-42), anti-CD9 (ThermoFisher, cat# MA1-80307) antibodies at a concentration of 8 nM overnight, and then diluted 1000-fold in PBS 0.22 µm buffer before data collecting. Samples were measured on 96-well U bottom multiwell plates (Sarstedt) using the plate reader of the equipment. The samples were then analyzed with flowJo dongle v10.8.

#### Mass spectrometry analysis of EV cargo

Mass spectrometry analysis was performed by the Clinical Proteomics Mass Spectrometry facility, Karolinska Institutet/Science for Life Laboratory. Three extracellular vesicle samples collected and purified as mentioned above from pig bladder micrograft cultures were analysed, by label free quantification. To each approximately 33 µl of sample, 100 µl of Lysis buffer was added (4% SDS, 50 mM HEPES pH 7.6, 1 mM DTT). Protein digestion (trypsin, sequencing grade modified, Pierce) was performed using a modified protocol for SP3 protein clean up followed by SP3 peptide clean up^48^. Each sample was separated using a Thermo Scientific Dionex nano LC-system in a 3 h 5–40% ACN gradient coupled to Thermo Scientific High Field QExactive. All searches were done against *Sus scrofa* Uniprot database, using the core facility proteomics workflow (https://github.com/lehtiolab/ddamsproteomics vs2.7, SciLifeLab KI, SE). Protein false discovery rates were calculated using the picked-FDR method using gene symbols as protein groups and limited to 1% FDR^49^. A complete description of the identified proteins from the mass spectrometry can be found in Supplementary Table S1.

#### Proteome data analysis

The set of identified proteins were preprocessed using custom Python and R-scripts. Firstly, we excluded proteins not found in all 3 samples. Next the protein levels were normalized using standard normal variate scaling (SNV), allowing the comparations between samples. To control for contaminants, we excluded proteins with abnormally high expression (2*absolute normalized median expression levels). The remaining proteins were thereafter analyzed using the web interfaces g:Profiler and the Global Core Biodata Resource Panther to mine various kinds of biological data resources including gene ontology (GO) terms and Reactome pathways^31,32^. Gene ontologies cut off were FDR less 0.05, p-value less than 0.05, and the cut-off criterion of significant enriched pathways was p-value less than 0.05. The resulting enriched terms were ranked by a p-value based on g:GOSt, a g:Profiler multiple testing correction. STRING wed data base was used to visualize proteins protein interactions in the EV proteins.

### Statistical analyses

Unless stated otherwise, numbers are presented in means with standard deviations, and summarized results are presented in either bar charts or dot plots. When applicable, comparison of continuous variables was done with two-tailed independent t-test, with p < 0.05 considered as statistically significant. Analyses were carried out in Microsoft Excel© (Microsoft Corporation 2016) and R© (R Core Team (2013). R: A language and environment for statistical computing. R Foundation for Statistical Computing, Vienna, Austria. URL http://www.R-project.org/).

### Ethical permissions

The tissues extracted for this study were obtained postmortem from pigs previously used in surgical training. The pigs were euthanized after the surgical procedure in accordance with European legislations for laboratory animals, and therefore further ethical permission for this in vitro study was not required.

### Supplementary Information

Supplementary Figure 1.

Supplementary Legends.

Supplementary Table 1.

## Data Availability

Any data not presented in the publication can be made available upon request to the corresponding author.
