# Supplementary figures and images for: Insights into cellular behavior and micromolecular communication in urothelial micrografts
Source: Sci Rep. 2023 Aug 21;13:13589. doi: 10.1038/s41598-023-40049-0 (PMC10442416; doi:10.1038/s41598-023-40049-0)

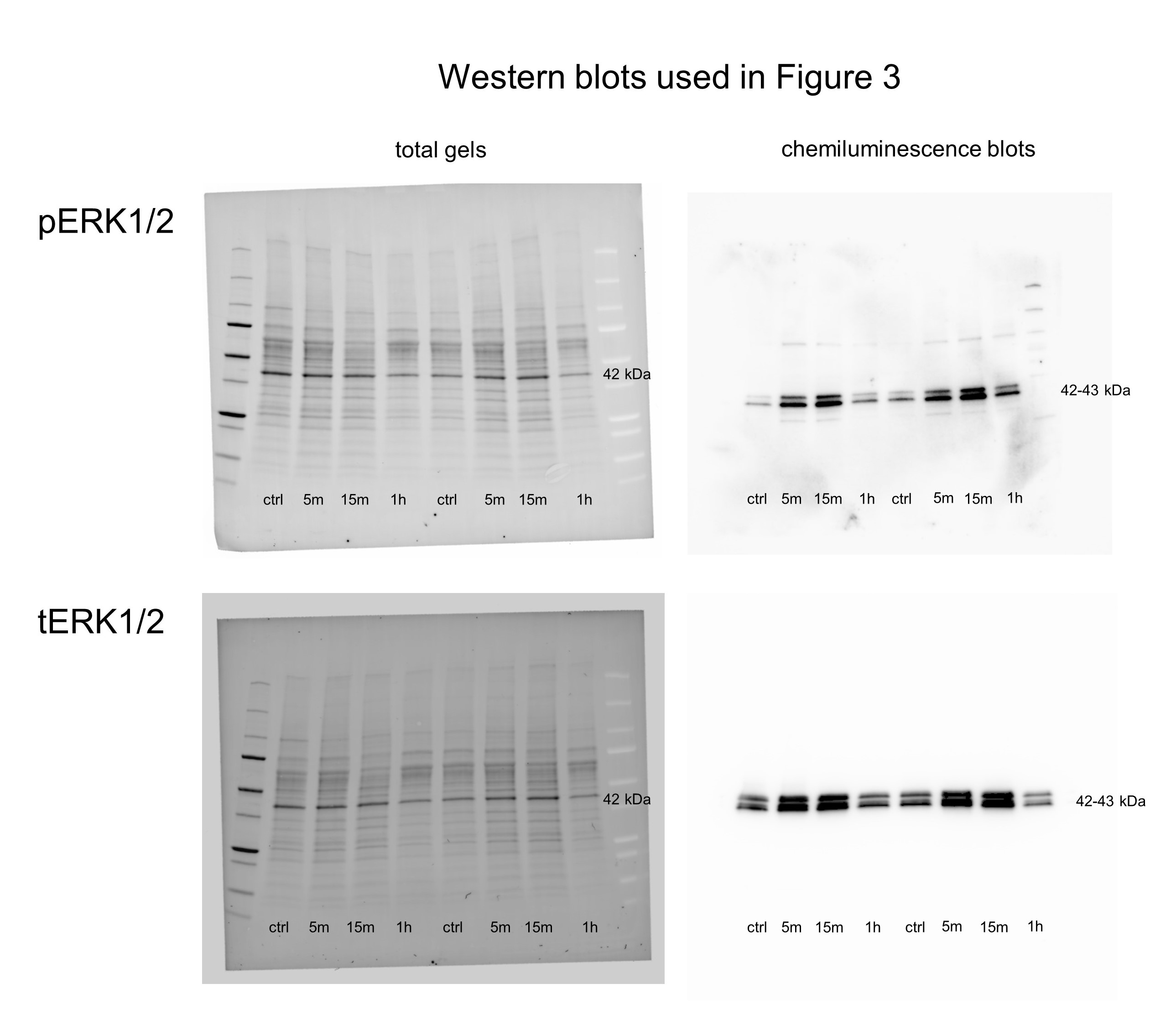

Supplement: Supplementary file 1 — Supplementary Figure 1. [file 41598_2023_40049_MOESM1_ESM.jpg]
