# Supplementary material for: Insights into cellular behavior and micromolecular communication in urothelial micrografts
Source: Sci Rep. 2023 Aug 21;13:13589. doi: 10.1038/s41598-023-40049-0 (PMC10442416; doi:10.1038/s41598-023-40049-0)
Supplement: Supplementary file 2 — Supplementary Legends. [file 41598_2023_40049_MOESM2_ESM.docx]

Legends for supplementary files

**Supplementary figure 1.**

Complete Western blots used in figure 3.

**Supplementary table 1.**

Complete list of identified EV proteins used for gProfiler and STRING pathway analyses.
